# Platelet-Activating Biominerals Enhanced Injectable Hydrogels With Superior Bioactivity for Bone Regeneration

**DOI:** 10.3389/fbioe.2022.826855

**Published:** 2022-03-07

**Authors:** Xin Chen, Jiajun Yan, Yingying Jiang, Yunshan Fan, Zhengran Ying, Shuo Tan, Zhi Zhou, Junjian Liu, Feng Chen, Shisheng He

**Affiliations:** ^1^ Department of Orthopedic, Spinal Pain Research Institute, Shanghai Tenth People’s Hospital, Tongji University School of Medicine, Shanghai, China; ^2^ National Engineering Research Center for Nanotechnology, Shanghai, China

**Keywords:** calcium phosphate, biominerals, hydrogels, platelets, bone regeneration

## Abstract

Refractory bone fracture, which is difficult to be treated, is a common clinical disease. Taking inspiration from the natural process of bone regeneration, we provide a biomimetic strategy to develop a new injectable biomaterial for repairing bone defects, which is mainly composed of platelets, fibrins, and biominerals. Biomineral nanoparticles (EACPNs) with an amorphous phase are prepared by an enzyme-catalyzed route and display a platelet-activating property. The composite hydrogel (EPH) of EACPNs, fibrins, and platelets is injectable, and has similar chemical properties to natural materials in bone regeneration. The dried EPH samples display a highly porous structure, which would be favorable for cell attachment and growth. The results from *in vitro* studies indicate that EPH has high biocompatibility and superior bioactivity in promoting the osteogenic differentiation of rat bone marrow stem cells (rBMSCs). Furthermore, the results from *in vivo* studies clearly indicate that EPH can induce the formation of new collagen and vessels in the defect area, thus leading to faster regeneration of bone defects at 2 weeks. Our study provides a strategy for designing new biomimetic materials, which may be favorable in the treatment of refractory bone fracture.

## Introduction

Refractory bone fracture disease is a constant problem among orthopedic patients in hospitals that leads to a serious impact on the health and quality of life. Biomaterials with high biocompatibility and bioactivity that promote bone regeneration will be promising in the treatment of refractory bone fractures and can be implanted into defect areas by a simple injection (Xu et al., 2020). However, it is still a critical problem to prepare such high-performance bone repair materials in orthopedic research fields. Fortunately, the natural bone regeneration process has provided great inspiration for developing such high-performance biomaterials.

The natural healing process of bone defects is a multistep process involving blood and various cells (e.g., immunological cells, osteoclasts, fibroblasts, and osteoblasts). In the early stage of bone regeneration, a hematoma is formed at the bone defect site by the coagulation of blood, and then the fibroblasts penetrate into the defect area to build a fibrous callus (De Pascale et al., 2015). The formation of hematoma plays an essential role. The activated platelets in the hematoma release a variety of growth factors and cytokines to promote tissue regeneration, including vascular endothelial growth factor (VEGF), fibroblast growth factor (FGF), platelet-derived growth factor (PDGF), and transforming growth factor β (TGF-β) (Ruggeri, 2002; Giusti et al., 2020). Moreover, the fibrinogen in plasma can transform into a tightly packed gel with a network microstructure, which can combine with the aforementioned growth factors to enhance tissue regeneration (Boswell et al., 2012; Mussano et al., 2016). The fibrinogen/platelet gels promote cell proliferation and induce the deposition of a new extracellular matrix (ECM), which is beneficial for the repair of damaged bone tissues (Aravamudhan et al., 2013; Shiu et al., 2014). Then, over many weeks, osteoblasts gradually transform the callus into a bone tissue through a biomineralization process.

Biomineralization is a complex process that produces heterogeneous minerals, leading to the hardening or stiffening of mineralized tissues in vertebrates (Wegst et al., 2015). The biomineralization process in mineralized tissues is usually regulated by osteoblasts, various molecules, and inorganic ions. Osteoblasts not only produce the collagenous extracellular matrix (ECM) but also secrete calcium phosphate (ACP) nanogranules, which are generated in mitochondria and further induce the subsequent mineralization of collagen to form the hierarchical structure of a bone (Martin and Matthews, 1970; Sutfin et al., 1971; Sayegh et al., 1974; Landis et al., 1993; Mahamid et al., 2010; Boonrungsiman et al., 2012; Niu et al., 2017; Lotsari et al., 2018; Reznikov et al., 2018). Due to the similar chemical properties to inorganic constituents of bone, synthetic calcium phosphate has been prepared and widely used in bone regeneration. Calcium phosphate-based biomaterials can promote bone repair (Kim et al., 2017; González Díaz et al., 2018) by supporting mineral crystal growth on the ECM and then promoting bone mineralization (Zhai et al., 2010; Wang et al., 2012; Wang et al., 2019).

Through the understanding of the bone formation process, we think that the combined use of calcium phosphate-based biominerals with natural constituents in bone regeneration may provide a new strategy to design functional biomaterials. Our previous work reported an enzymatic strategy to synthesize biominerals of amorphous calcium phosphate nanoparticles (EACPNs), which can promote the proliferation and osteogenic differentiation of hBMSCs (Jiang et al., 2018). The EACPNs may affect the activation of platelets and further lead to an acceleration of bone regeneration. However, there is no report on the effect of EACPNs on platelets and their combined use in bone regeneration, which will be significant for the design and construction of new biomaterials.

Herein, we developed a strategy to prepare injectable composite hydrogels for bone defect repair by combining the use of EACBN biominerals and platelets. EACPNs have been prepared with an enzyme-catalyzed route and display a platelet-activating property. *In vitro* studies have indicated that the composite hydrogel has high cell biocompatibility and superior bioactivity in inducing osteogenic differentiation of stem cells. Moreover, the *in vivo* experiment clearly shows the high performance of this composite hydrogel in promoting bone regeneration.

## Experimental

### Materials

The adenosine 5′-triphosphate disodium salt hydrate (Na_2_ATP) and alkaline phosphatase (ALP) powders were purchased from Sigma–Aldrich (United States). Fibrin sealant kits were obtained from Shanghai RAAS Blood Products Co., Ltd. (China). Other chemicals used in material preparation were obtained from Aladdin Industrial Corporation (Shanghai, China) if there was no special declaration. All the chemicals were used as received without further purification.

### Enzyme-Catalyzed Synthesis of Biominerals

EACPNs were prepared *via* an enzymatic reaction strategy reported in our previous work (Jiang et al., 2018). Briefly, CaCl_2_ (66.0 mg) was dissolved into deionized water (30.0 ml), and then aqueous solution (30.0 ml) containing Na_2_ATP (110.0 mg) was added drop-wise into the above Ca^2+^ solution under magnetic stirring. Then, 6.0 µl of alkaline phosphatase solution (6.0 U ml^−1^) was added to the above mixed reactive solution. The whole reaction was conducted for 3 h in a constant temperature bath (37°C), and the pH value was maintained at 8.0–8.5 by adding NaOH solution (0.2 M) throughout the reaction process. Finally, the products were collected by centrifugation and separately washed with DI water and ethanol three times. Then, the samples were freeze-dried for storage or further use.

### Interaction Between Platelets and EACBN Biominerals

Platelets were derived from commercial platelet solutions, which were supplied by the Shanghai Blood Center (Shanghai, China). Typically, 4 ml of platelet solution was separated by centrifugation at 8,000 rpm min^−1^ for 3 min at room temperature, and then 3 ml of the supernatant was subsequently removed. The remaining plasma and platelets that were concentrated 4 times were mixed and collected for further use. Then, EACPNs (10 mg/ml) were mixed with the above platelet solution, and after a certain period of reaction at 37°C, their fluidity was observed to determine the time for the material to activate platelets and promote coagulation. Platelets in the resting state were used as a control. In addition, we observed the morphology of platelets interacting with EACPNs by scanning electron microscopy (SEM).

### Preparation of EACPN-Platelet Injectable Hydrogels

In the preparation of the hydrogel, we used two reactive solutions that were put into two different syringes. A reaction can occur when these two solutions are mixed at the same time, and then the solutions turn into hydrogels. Typically, 400.0 μg of EACPNs was dispersed into 20.0 μl of fibrinogen solution (40 mg/ml), and then 10 µl of 4 times concentrated platelet solution was added into the above solution under magnetic stirring to obtain Solution A. Fibrinogen was dissolved according to the manufacturer’s instructions. Thereafter, 20 μl of thrombin solution with a concentration of 500 IU/ml was prepared as solution B. Finally, when Solution A and Solution B were mixed, an EACBN–platelet composite hydrogel (EPH) was obtained. The control sample of platelet hydrogel (PH) was prepared in the same way without adding EACPNs. Moreover, the other control sample of fibrinogen hydrogel (FH) was prepared by directly mixing 20 μl of fibrinogen solution with 20 μl of thrombin solution without adding EACPNs or platelets. The as-prepared EPH, PH, and FH were further freeze-dried *via* lyophilization for further characterization.

### Swelling Ratio of Materials

To study the influence of EACPNs on the swelling ratio of samples, the different samples were injected into round molds, and then cylindrical samples (5 mm in diameter and 5 mm in height) were obtained. All the samples were freeze-dried and immersed in PBS solution (37°C 120 rpm) after being washed with PBS solution and weighed separately. The dry weight of each sample was Wd. After immersing in the PBS solution for different times, each sample was weighed again to obtain its wet weight (Ww).

The formulation to acquire the swelling ratio is as follows:

Swelling ratio=(Ww−Wd)/Wd ·100%.

### Material Characterization

Mechanical strength is important for the regeneration of defective bones. To acquire the mechanical properties of the samples, different cylindrical samples (5 mm in diameter and 5 mm in height) were prepared and compressed at a constant speed of 5 mm min^−1^. The accurate diameter and height of each sample were recorded before testing. At least three samples were measured to obtain the mechanical properties of the samples.

### Cell Activity

Rat bone marrow mesenchymal stem cells (rBMSCs) were derived from the femurs of Sprague–Dawley (SD) rats and were provided by the Shanghai SLAC Experimental Animal Center (Shanghai, China). The cells were cultured in Dulbecco’s minimum essential medium (DMEM, Sigma Life Science) supplemented with 10% fetal bovine serum (FBS) and 1% penicillin–streptomycin (PS) at 37°C under a 5% CO_2_ humidified atmosphere. All the following tests were conducted using rBMSCs from the third to the fifth passages. The freeze-dried gels were irradiated in ultraviolet light for 60 min, immersed in 1,000 µl of raw DMEM at room temperature for 24 h, and then centrifuged at 8,000 rpm to extract the supernatant. The supernatant extracted from freeze-dried EPH was diluted with DMEM for different ratios. Then, rBMSCs were co-cultured with the extracted supernatant supplemented with 10% FBS and 1% PS in a 96-well plate with a cell concentration of 3,000 cells per well for 48 h to evaluate the cell viability of the samples with a CCK-8 assay (Beyotime Biotechnology, Shanghai, China).

In addition, to evaluate whether the ACPN–platelet composite hydrogel has the ability to induce chemotaxis of cells, human umbilical vein endothelial cells (HUVECs, Cell Bank of the Chinese Academy of Sciences, Shanghai, China) were seeded into a 6-well plate at a concentration of 2×10^4^ cells per well and co-cultured with the extracted supernatant of FH, PH, and EPH (at 24 h). Then, the cells were preserved and stained with crystal violet staining solution (Beyotime, China) and observed under an inverted fluorescence microscope (Leica, Germany).

### Alkaline Phosphatase (ALP) Staining

To assess the ALP activity of the supernatant-treated rBMSCs at Days 7 and 14, the co-cultured cells were washed with PBS, fixed in 4% paraformaldehyde for 20 min and stained with an Alkaline Phosphatase Kit (Beyotime Biotechnology, Shanghai, China). Optical images were taken using a Leica inverted microscope.

### Real-Time Quantitative PCR

The rBMSCs co-cultured with the supernatant in a 6-well plate were washed with PBS and collected at 14 days. Then, the total RNA in rBMSCs was isolated by TRIzol reagent (Thermo Fisher Scientific, American). The TaKaRa reagents PrimeScript RT Master Mix and SYBR Premix Ex Taq were used for RNA reverse transcription and RT–qPCR, respectively. The expression of osteocalcin (OCN), osteopontin (OPN), and Runx2 was measured. The data were normalized to the expression of glyceraldehyde 3-phosphate dehydrogenase and analyzed by the control Ct (2^−ΔΔCt^) method.

### *In Vivo* Experiment

The animal experiment was performed according to the plan approved by the Ethics Committee of Shanghai Tenth People’s Hospital. Twenty 6-week-old male Sprague–Dawley (SD) rats were provided by the Shanghai SLAC Experimental Animal Center and were randomly divided into 4 groups. All rats were given food and water in the absence of specific pathogens (12 h light/dark cycle, 23°C). The operation was performed aseptically on the right femur. After inhaling isoflurane anesthesia, the rat’s surgical area was scraped and disinfected with iodophor. The skin was cut by a scalpel and peeled off from the muscle space to the bone surface. Then, a unilateral bone penetrating the defect with a diameter of 2.4 mm was made at the middle and lower parts of the femur using a tungsten steel ball drill. Later, the prepared gels were implanted, and the muscular layer and skin were sutured. The bone defects that were unfilled were set as the blank group, and bone defects that were filled by injection with FH, PH, and EPH hydrogels were prepared under aseptic conditions.

Two weeks later, all the rats were euthanized, and the femurs with defects were fixed with 4% paraformaldehyde for 48 h at 4°C. The femurs were scanned by micro-CT (SkyScan1276, Bruker Corporation, United States) and reconstructed *via* SkyScan NRecon software. Indices such as bone volume/tissue volume (BV/TV), trabecular thickness (Tb.Th), number (Tb.N), and separation (Tb.Sp) were analyzed using SkyScan CT-Analyzer software to evaluate the effect of bone defect repair. For the hematoxylin–eosin and Masson staining, the fixed femur samples were decalcified with EDTA solution for 1 month. The decalcified specimens were embedded in paraffin, and the sections were stained with H&E and Masson. All chemicals were purchased from Wuhan Service Bio Technology Co., Ltd., China. Representative images were taken by a Leica vertical microscope.

### Statistical Analysis

The data were analyzed *via* SPSS 24.0 software. One-way ANOVA and *t*-tests were used to compare between groups. All data are expressed as the average ±SD. Each experiment contained at least three repeats. Only when *p < 0.05, **p < 0.01, and ***p < 0.001, the difference was regarded as statistically significant.

## Results and Discussion

### Preparation and Characterization of EACPNs

**FIGURE 1:**
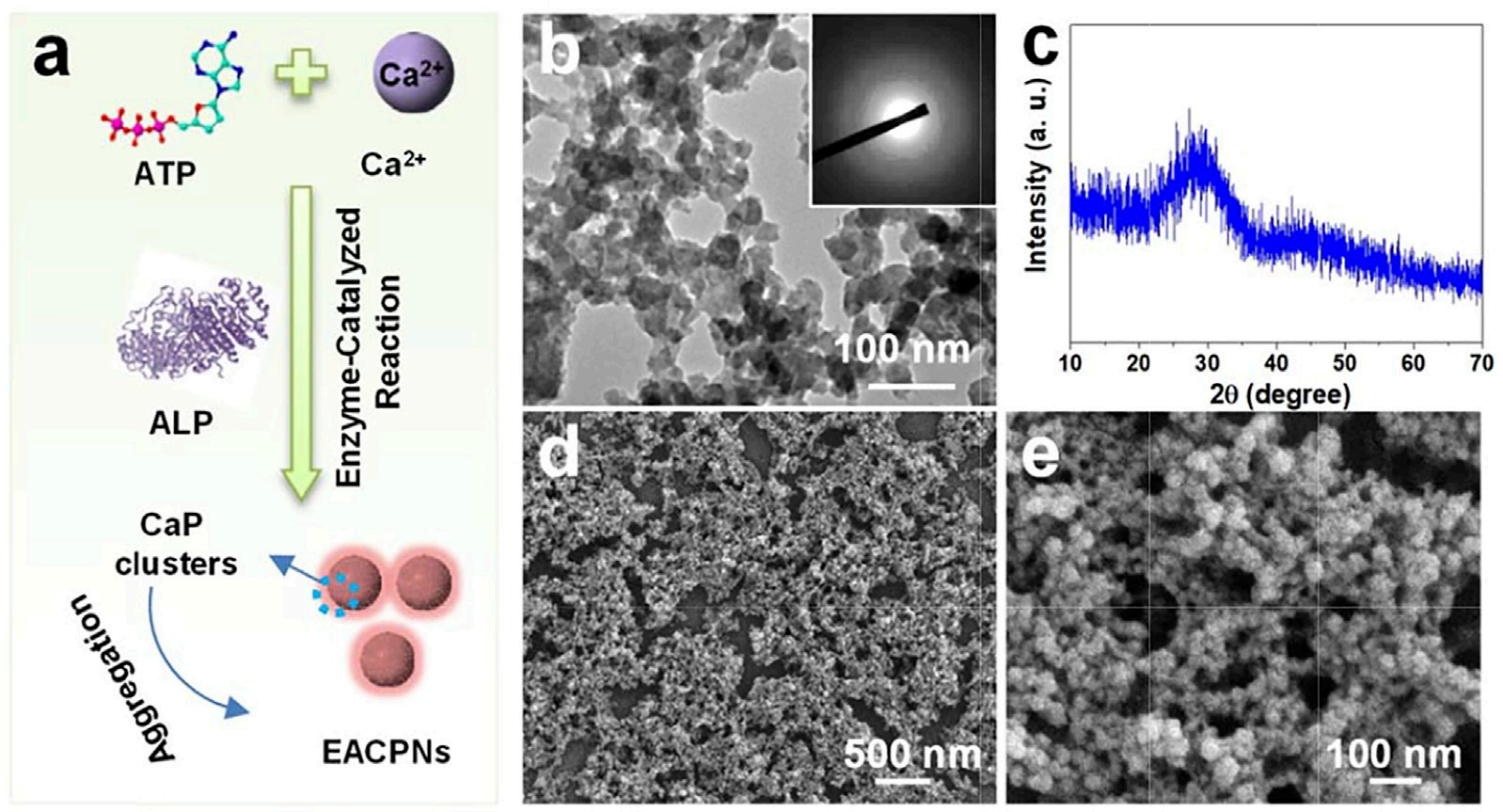
Schematic illustration of preparation and characterization of EACPNs. **(A)** The enzyme-catalyzed reaction route for the preparation of EACPNs; characterization results of TEM **(B)**, XRD **(C)**, and SEM **(D,E)** of EACPNs.

Figure 1A displays the route of the enzyme-catalyzed reaction for the preparation of EACPNs. Figure 1B shows transmission electron microscopy (TEM) micrographs of EACPNs, in which the samples display an amorphous structure of nanoparticles with a diameter of 20–50 nm. The selected-area electron diffraction (SAED) pattern (insert) also shows a typical pattern of an amorphous structure. These results agree well with the XRD pattern of the EACPNs in Figure 1C. Furthermore, the SEM micrographs (Figures 1D,E) of EACPNs display a spherical morphology, which is consistent with the TEM results.

In the reaction process, the ALP enzyme acts as a cleaver to hydrolyze phosphate ions, which react with calcium ions to form EACPNs from ATP molecules. After the enzyme-catalyzed reaction, some ATP molecules can transform to ADP and AMP. The molecules of ATP/ADP/AMP can interact with the calcium ions of mineral clusters and inhibit the crystallization of these clusters. Then, these mineral clusters form amorphous aggregates of EACPNs.

### The Interaction Between EACPNs and Platelets

**FIGURE 2:**
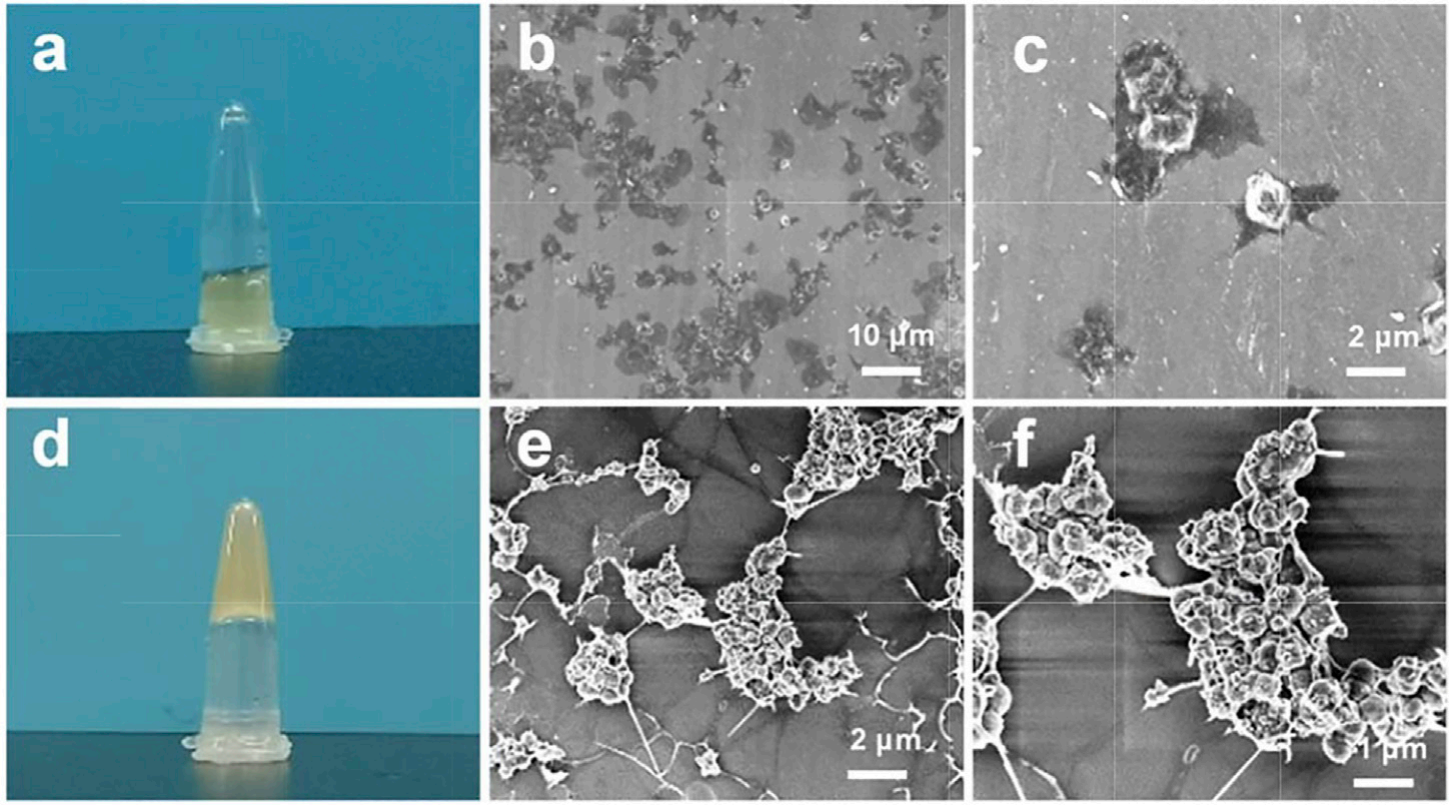
Digital photographs and SEM micrographs of platelets in resting states **(A–C)** and EACPN-treated platelets **(D–F)**.

In an *in vitro* study of the interaction between EACPNs and platelets, we found that EACPNs and platelets have a significant interaction that can promote platelet activation and coagulation (Figure 2). When EACPNs are incubated with platelets for approximately 2 min, we can observe that the originally flowing platelet solution loses its fluidity and can be stably fixed on the bottom of the inverted centrifuge tube (Figure 2D). SEM micrographs of platelets in the resting state and EACBN-treated platelets are shown in Figure 2. The platelets in the resting state showed a smooth discoid shape (Figures 2B,C). In contrast, after treatment with EACPNs, there was a significant change in the morphologies of platelets, which displayed a typical activated state with many channels of the open canalicular system (OCS) (Figures 2D,E).

We think that the activation of platelets is related to the large amount of ADP molecules in EACPNs. In our previous study, we revealed the content of ADP molecules in EACPNs by a high-performance liquid chromatography (HPLC) method, which indicated that there is approximately 0.15 g of ADP molecules in each gram of EACPNs (Jiang et al., 2018). It has been reported that ADP, as a soluble agonist released by activated cells, can trigger platelet activation through GPCRs, thereby promoting coagulation formation (Yun et al., 2016).

As fragments of blood cells, platelets (in an activated state) play essential roles in defense against excessive blood loss and wound repair. For example, in the bone regeneration process, platelets play a critical role in the hematoma formation process and in blood coagulation, which provide the supporting sites and environment for the growth of different cells. Activated platelets also release a variety of growth factors and cytokines to promote the proliferation or differentiation of bone repair-related cells. Platelets have been widely used in promoting tissue regeneration, including stomatology (Attia et al., 2020), maxillofacial surgery (Chou et al., 2020), trauma, and orthopedics (Hohmann et al., 2021). Therefore, we believe that the combination of EACPNs and platelets is a potential choice in developing bioactive materials for bone defect repair.

### Characterization of EACPN–Platelet Hydrogels

After mixing solutions of EACPNs, platelets, and fibrinogen/thrombin, a hydrogel of EPH was obtained. The natural coagulation of blood contains much fibrinogen from blood. The platelet solution is concentrated and short of fibrinogen. Therefore, we have added fibrinogen to the preparation process of EPH. The hydrogels of PH (without EACPNs) and FH (without platelets) were used as control samples. The as-prepared FH, PH, and EPH were freeze-dried *via* lyophilization for further characterization.

**FIGURE 3:**
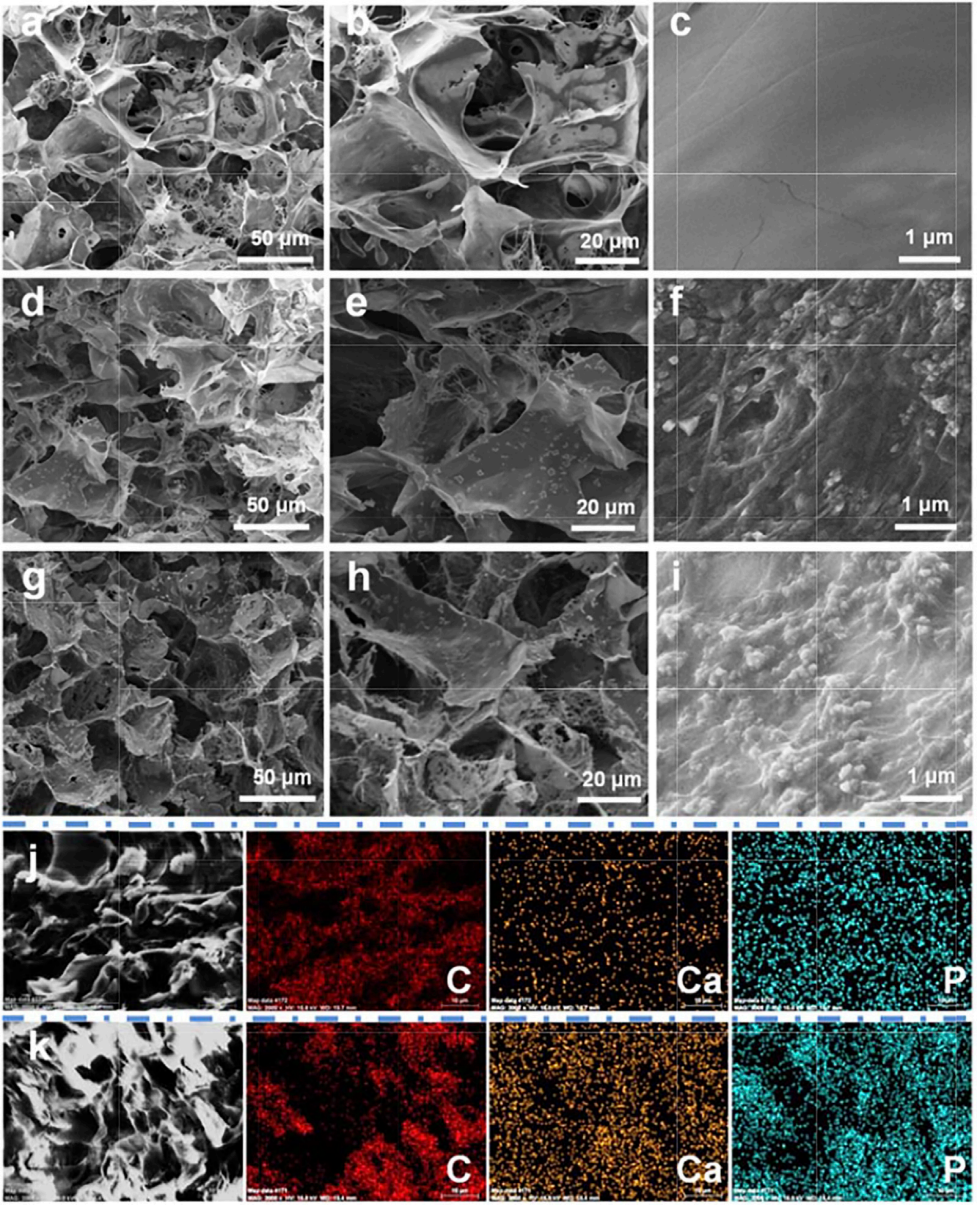
SEM micrograph and element distribution mappings of EACPN–platelet hydrogels and control samples. **(A–I)** SEM micrographs of freeze-dried hydrogels of FH **(A–C)**, PH **(D–F)**, and EPH **(G–I)**; **(J,K)** C, Ca, and P distribution mappings of selected areas of control sample of PH **(J)** and EPH **(K)**.

Figure 3 displays the SEM micrographs and element distribution of EPH and control samples. As shown in Figures 3A–I, typical 3D structures with interconnected micropores were observed in all samples of dried FH, PH, and EPH hydrogels. There is no significant difference in the morphologies with low magnification among these three samples, which have large pores with diameters of 100–500 μm. However, in the SEM images with high magnification, we find that there are significant differences among the three samples. On the wall of the porous structure, the FH sample has a smooth surface structure. In contrast, the PH and EPH samples have rough surfaces with a large number of activated platelets.

The EACPNs are small and can be encased in the wall of the porous structure, which cannot be clearly observed in the SEM micrographs. Therefore, the results of element mappings are given to illustrate the distribution of EACPNs. Figures 3I,K display the element distributions of C, Ca, and P in the selected areas of the control samples of PH and EPH, which show that the contents of calcium and phosphorus in EPH are significantly higher than those in PH. The distribution of calcium and phosphorus is consistent with the distribution of carbon, which indicates the successful incorporation of EACPNs in the EPH sample. Compared with the control sample of PH, the amount of calcium and phosphate increased from 0.51 to 0.56 % to 2.71 and 2.80 %, respectively. Moreover, the results of reconstructed micro-CT images of dried hydrogels of PH and EPH are given in Supplementary Figure S1, which display the difference in the mineral density of the two samples. The images also perfectly display the internal interconnected structure inside the EPH sample. The obvious difference in mineral density between the PH and EPH samples can be well explained by the addition of EACPNs to the EPH sample.

Furthermore, the results of the swelling ratio and mechanical properties of the three materials are also given in Supplementary Figures S2A,B. The image of the swelling ratio shows that dried EPH could absorb the surrounding liquid in a short time and reach equilibrium in a shorter time than the samples of dried samples of FH and PH. Moreover, from the results of mechanical tests, a stronger mechanical bearing capacity was obtained in the EPH group than in the other two groups. The results may be explained by the addition of EACPNs into the EPH samples, which may affect the mechanical properties by a reinforcement effect. These results indicate that the addition of EACPNs can affect the physical properties of materials.

The FTIR spectra of EACPNs, FH, PH, and EPH are shown in Supplementary Figure S3. The broad absorption bands in the spectra of all samples at 3,200–3,600 cm^−1^ originate from the water molecules adsorbed by the samples. In the spectra of EACPNs and EPH, the absorption peaks at approximately 1,109 cm^−1^ and 563 cm^−1^ are higher than those of FH and PH, which can be assigned to the asymmetric stretching vibration of PO_4_^3−^. The absorption peaks at approximately 1,649 cm^−1^ originate from adenosine or other organic molecules from platelets, which correspond to the stretching mode of C=N. These FTIR spectra indicate the components of the samples, which are consistent with the above element mapping results.

### *In Vitro* Biocompatibility and Bioactivity of EPH

**FIGURE 4:**
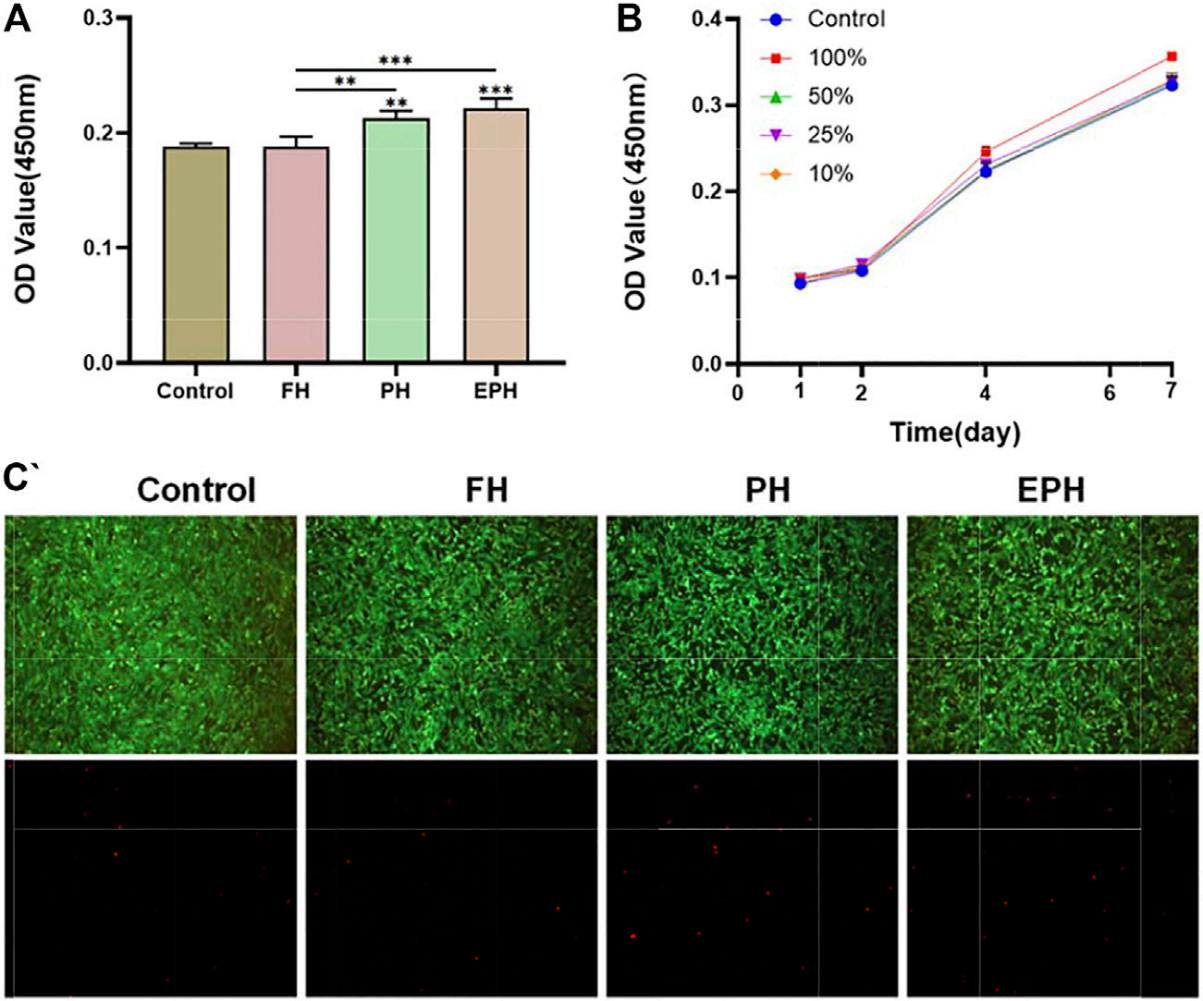
**(A)** Cell viability of rBMSCs co-cultured with the corresponding extracted supernatant from FH, PH, and EPH for 48 h. **(B)** The proliferation curve of rBMSCs treated with a series of diluted supernatant of EPH. **(C)** Fluorescence images of dead/live cell staining after incubation with extracted supernatant of FH, PH, and EPH. Fluorescence microscopy images of four groups stained with calcein–AM/PI.

To further evaluate the *in vitro* biocompatibility of FH, PH, and EPH, rBMSCs were co-cultured with the extracted supernatant of hydrogel samples. As shown in Figure 4A, the OD values from the CCK-8 assay of the PH and EPH groups were higher than those of the control, and FH showed a similar value as the control. The results reveal the high biocompatibility of EPH, PH, and FH. Afterward, a series of dilutions of EPH supernatant were used to further explore the effect of EPH on the proliferation of rBMSCs. The results in Figure 4B show that all the groups cultured with supernatants show good proliferation curves. The proliferation rate of rBMSCs treated with 100% supernatant was slightly faster than that of rBMSCs in the other groups. Furthermore, there was no significant difference in the live/dead cell staining of the four groups, which also indicated the good biocompatibility of these hydrogels (Figure 4C).

**FIGURE 5:**
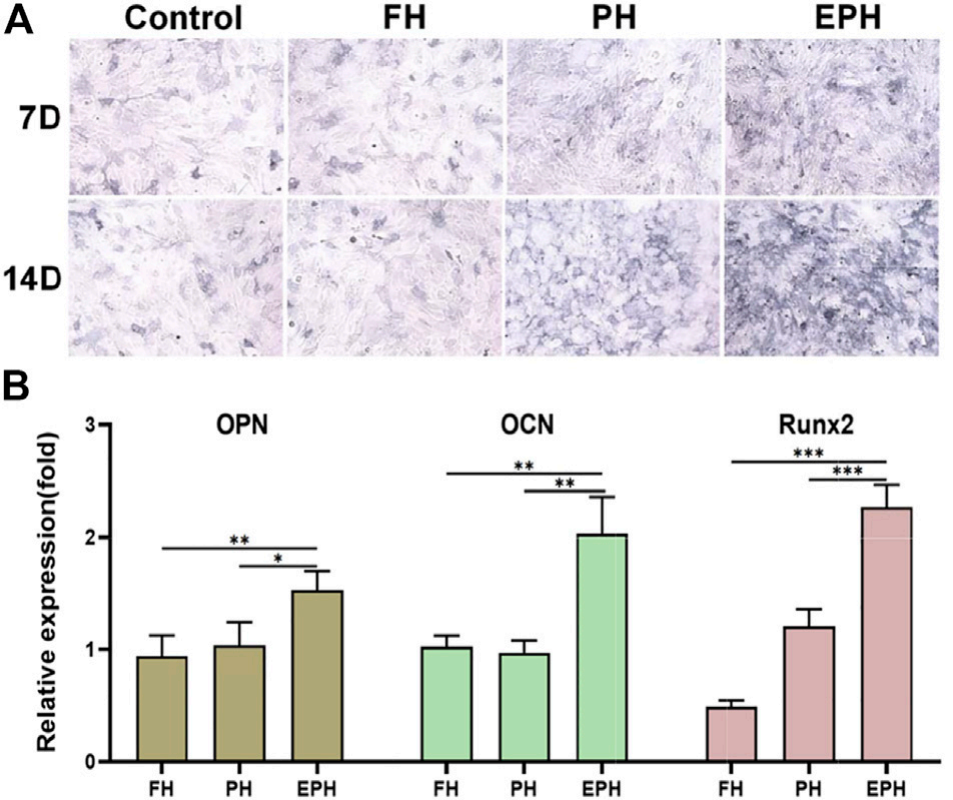
**(A)** ALP staining of rBMSCs incubated with the supernatant of hydrogels of FH, PH, and EPH for 7 and 14 days, respectively. **(B)** The expression of osteopontin (OPN), osteocalcin (OCN), Osterix, and Runx2 genes of rBMSCs incubated with the supernatant of FH, PH, and EPH for 14 days, respectively.

Thereafter, the effects of FH, PH, and EPH on the osteogenic differentiation of rBMSCs were studied. The results of alkaline phosphatase (ALP) staining are given in Figure 5A. The images show that the ALP activity of rBMSCs incubated with the supernatant of EPH was higher than that of the control and the other groups. Then, the total RNA of OPN, OCN, and Runx2 expressed in rBMSCs was also studied (Figure 5B). The results clearly indicated that the examined genes were obviously upregulated after treatment with the supernatant of EPH, which was obviously higher than that of FH and PH.

**FIGURE 6:**
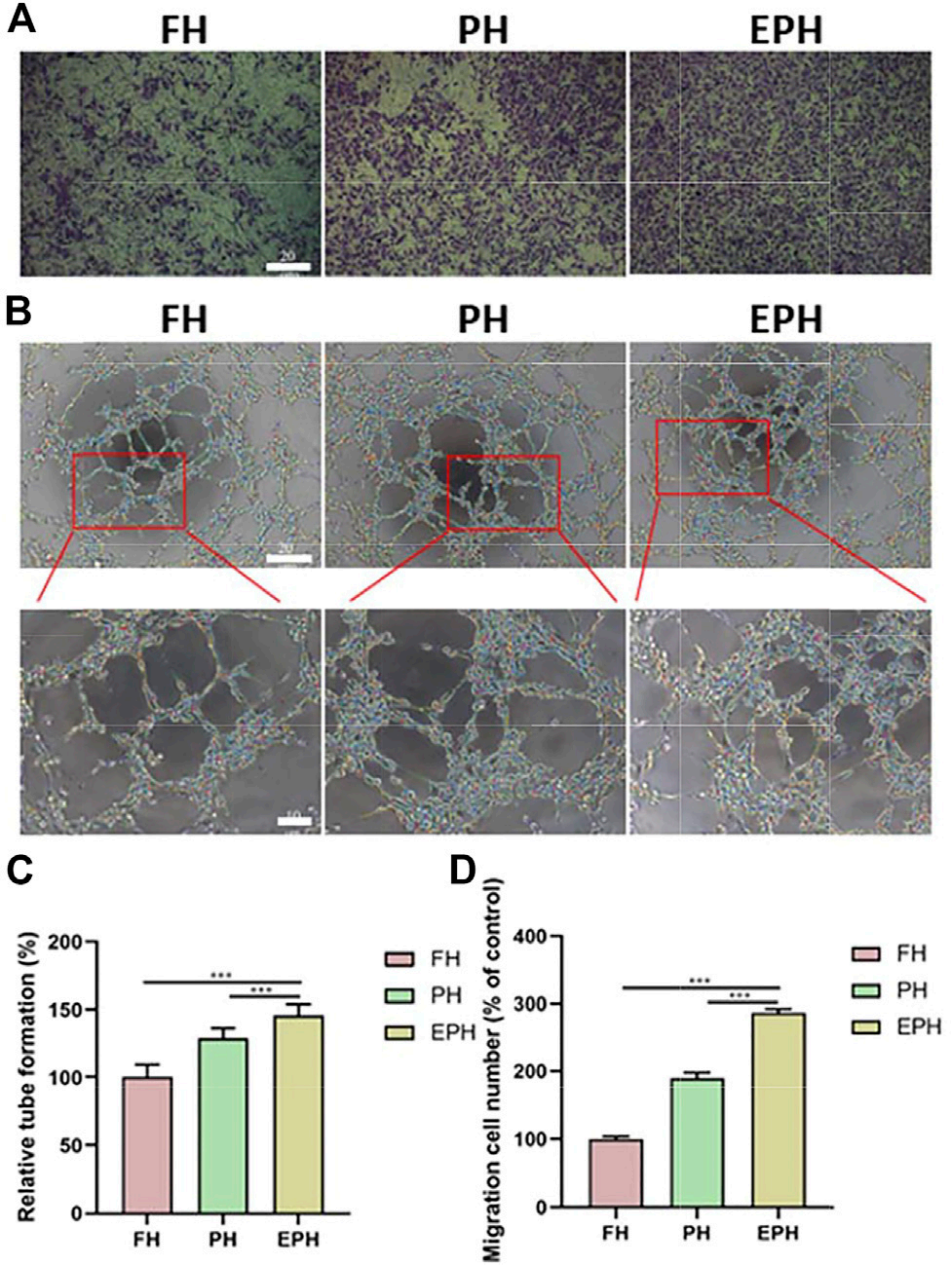
**(A)** Transwell migration assay of HUVECs incubated with the extracted supernatant of FH, PH, and EPH hydrogels for 24 h, respectively; **(B)** The tube formation of HUVECs incubated with the extracted supernatant of FH, PH, and EPH for 24 h, respectively. **(C)** Quantitative data of the formed tube in **(B)**; **(D)** Quantitative data of the migration cell number obtained from transwell assay.

In addition, we evaluated the effects of FH, PH, and EPH on the migration and tube formation properties of HUVECs. In Figures 6A,D, the images and analyzed data obtained from the transwell assay clearly show that the supernatant from PH and EPH can promote the migration of HUVECs compared with that from FH. Furthermore, we investigated the angiogenic capacity of HUVECs using *in vitro* tube formation experiments. As shown in Figures 6B,C, the images and analyzed data show that PH and EPH had significantly better effects on the tube formation ability of HUVECs than FH materials. This result can be easily understood because it is well known that platelets and the abundant growth factors in platelets may contribute to the migration, growth, and angiogenesis of vascular endothelial cells.

### *In Vivo* Studies of EPH in a Rat Defect Model

**FIGURE 7:**
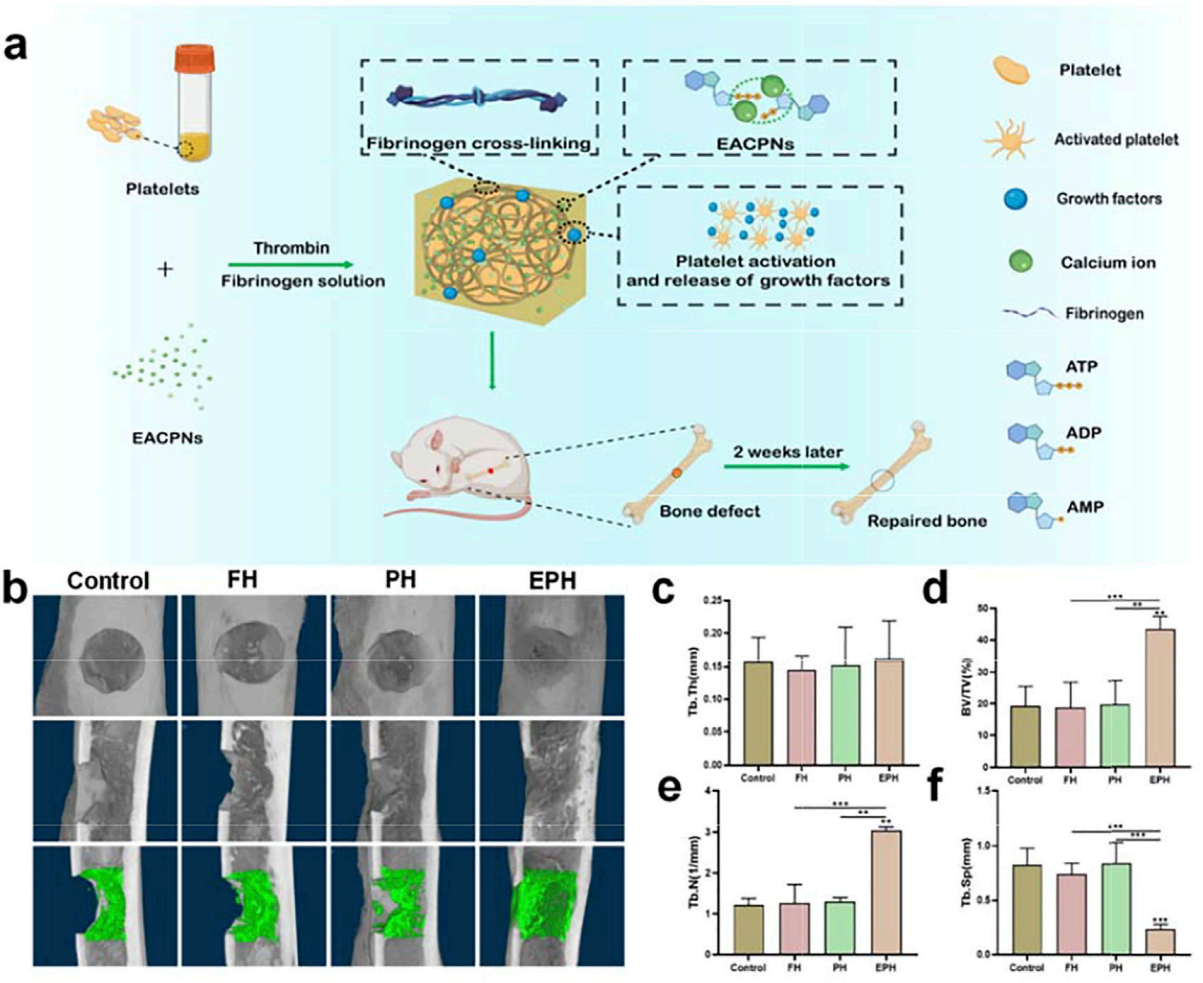
**(A)** Schematic illustration of the preparation and application of EPH and controls in the repair of an *in vivo* femoral defect model with a diameter of 2.4 mm; **(B)** reconstructed micro-CT images of the defects treated with EPH and controls for 2 weeks post-op (First row: front view; second row: sectional view; third row: regenerative areas highlighted from the sectional view); **(C–F)** the analysis results of the trabecular thickness (Tb.Th) **(C)**, bone volume density (BV/TV); and **(D)** trabecular number (Tb.N) **(E)** and trabecular separation (Tb.Sp) **(F)** of the defective areas after treated with the samples for 2 weeks.

The *in vivo* performance of EPH materials has been investigated using a rat defect model in the femoral bone with a diameter of 2.4 mm, which is a small defect that mimics bone fractures. Figure 7A displays a schematic illustration of the preparation and application of EPH in repairing an *in vivo* bone defect model. Figure 7B shows the results of reconstructed micro-CT images of the bone defects in the femoral bones of the rats after treatment with EPH and controls for 2 weeks. The images clearly display that there is more regenerative bone formed in the EPH group from both the front and sectional views. The regenerative bone in defect areas is highlighted and shown in the third row of Figure 7B. Obviously, EPH provides a significant promotion of the formation of new cortical and cancellous bone. Micro-CT quantitative analysis further confirmed that EPH has a superior capacity in promoting bone regeneration. However, a large difference was not observed among the four groups in trabecular thickness (Tb.Th) (Figure 7C), and it can be seen that the bone volume/tissue volume (BV/TV) in the EPH group increased from 20 to 43% compared with those of the other groups (Figure 7D). Moreover, the trabecular number (Tb.N) also significantly improved in the EPH group (Figure 7E), while the decreased trabecular septum (Tb.Sp) further indicated the improvement in bone mass after treatment with EPH (Figure 7F).

**FIGURE 8:**
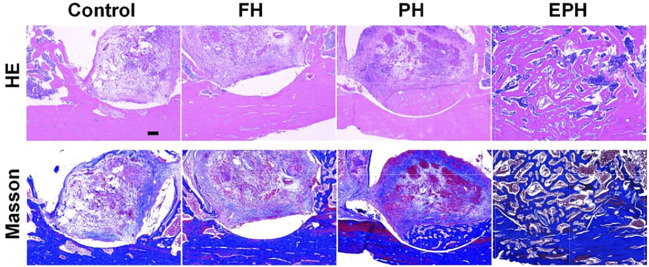
Optical images of histological samples with hematoxylin and eosin (H&E) staining and Masson’s trichrome staining. The scale bar in images is 200 µm.

Then, histological evaluation was performed by hematoxylin–eosin (H&E) staining and the Masson trichrome staining (Figure 8). As we can see from the front of the femur specimens, there is a small amount of new bone collagen and new vessels in the defect area of the blank control and FH groups. Moreover, the amount of bone collagen and the distribution were relatively improved in the PH group, and there were more new vessels. However, a large amount of continuous new bone collagen was found in the EPH group, showing better woven bone formation than the other three groups, and the bone defects were almost completely filled.

The excellent *in vivo* behavior of EPH as a bone repair material can be explained as follows: First, platelets and fibrinogen in EPH are natural, endogenous, degradable materials that can provide mechanical support for cell growth and attachment. Second, platelets release a large number of growth factors, which effectively induce chemotaxis of various cells and promote cell proliferation (El-Sharkawy et al., 2007). In addition, platelets and fibrinogen have the ability to increase vascular permeability and promote angiogenesis (Kubota et al., 2004). Finally, EACPNs combined in the scaffold can further induce the osteogenic differentiation of rBMSCs *via* a favorable interaction. Our previous study proved that EACPNs can enter cells by an endocytosis effect and can then cause autophagy and activate the AMPK pathway in BMSCs, which promotes the osteogenic differentiation of these cells (Jiang et al., 2018). The good performance of EPH in promoting bone defect repair is caused by the abovementioned factors, and it has good potential for future clinical application.

## Conclusion

In this study, a biomimetic hybrid hydrogel of EPH inspired by the formation of natural bone was designed and successfully prepared by using EACPNs, fibrin, and platelets. EACPNs, which have an amorphous structure and display a platelet-activating property, have been prepared by an enzyme-catalyzed route. After mixing with fibrin and platelets, the composite hydrogel of EPH with an injectable property is prepared, with excellent biocompatibility and bioactivity. EPH promoted the osteogenic differentiation of rBMSCs, which displayed the upregulated expression of OPN, OCN, Runx2, and Col I. Moreover, EPH displayed remarkable *in vivo* performance in promoting the regeneration of bone defects with a small size of 2.4 mm in 2 weeks by inducing the formation of new collagen and vessels in the defect area. Thus, this study provides a new strategy for constructing bioactive and injectable materials *via* a biomimetic strategy for effective bone regeneration.

## Data Availability

The original contributions presented in the study are included in the article/Supplementary Material, further inquiries can be directed to the corresponding authors.
