# Supplementary material for: Platelet-Activating Biominerals Enhanced Injectable Hydrogels With Superior Bioactivity for Bone Regeneration
Source: Front Bioeng Biotechnol. 2022 Mar 7;10:826855. doi: 10.3389/fbioe.2022.826855 (PMC8940219; doi:10.3389/fbioe.2022.826855)
Supplement: Supplementary file 1 [file DataSheet1.docx]

**Supporting Information**

**Platelet-Activating Biominerals Enhanced Injectable Hydrogels with Superior Bioactivity for Bone Regeneration**

Xin Chen,*^a,#^* Jiajun Yan,*^a,#^* Yingying Jiang,*^a,#^* Yunshan Fan,*^a^* Zhengran Ying,*^a^* Shuo Tan,*^a^* Zhi Zhou,*^a^* Junjian Liu,*^a^*^,^* Feng Chen,^a,b,^* and Shisheng He ^a,^*

a Department of Orthopedic, Spinal Pain Research Institute, Shanghai Tenth People’s Hospital, Tongji University School of Medicine, Shanghai 200072, P. R. China

b National Engineering Research Center for Nanotechnology, Shanghai, 200241, P. R. China

#These authors contributed equally to this work as co-first authors.

*corresponding author:

jjliu@tongji.edu.cn; fchen@tongji.edu.cn; tjhss7418@tongji.edu.cn

**Supporting Figures**


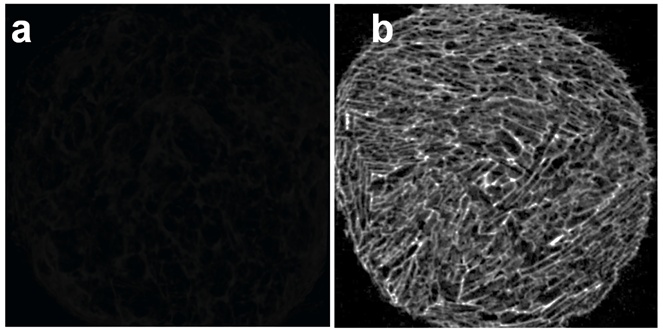


Figure S1. Reconstructed micro-CT images of (a) PH and (b) EPH with 3D structure;


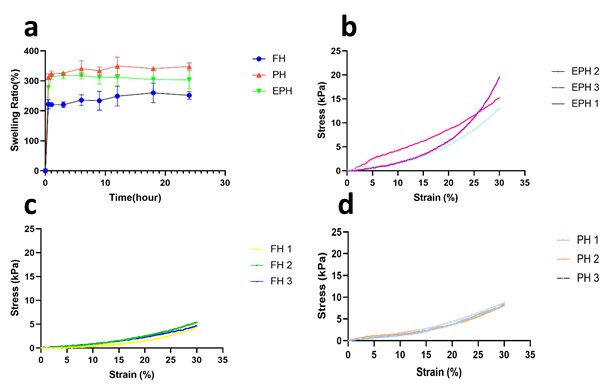


Figure S2. Swelling ration (a) and mechanical analysis (b-d) of FH、PH and EPH.

Figure S3. FTIR Spectra of freeze-dried EACPNs powders, FH, PH and EPH.

**Supporting Table**

**Table S1.**  The content of different elements in PH and EPH samples. The data is collected from the results of element distribution characterization in Figure 3. The unit of values in the table is at.%.

|  | Ca | P | C | O | N |
| --- | --- | --- | --- | --- | --- |
| PH | 0.51 | 0.56 | 59.76 | 21.15 | 12.62 |
| EPH | 2.71 | 2.80 | 57.07 | 21.21 | 10.92 |
